# A tool to automatically design multiplex PCR primer pairs for specific targets using diverse templates

**DOI:** 10.1038/s41598-023-43825-0

**Published:** 2023-09-30

**Authors:** Lin Yang, Feng Ding, Qiang Lin, Junhua Xie, Wei Fan, Fangyin Dai, Peng Cui, Wanfei Liu

**Affiliations:** 1 Shenzhen Branch, Guangdong Laboratory of Lingnan Modern Agriculture, Genome Analysis Laboratory of the Ministry of Agriculture and Rural Affairs, Agricultural Genomics Institute at Shenzhen, Chinese Academy of Agricultural Sciences, Shenzhen, 518120 China; 2 State Key Laboratory of Resource Insects, Institute of Sericulture and Systems Biology, Southwest University, Chongqing, 400715 China; 3 Shenzhen National Clinical Research Center for Infectious Diseases, No. 29, Bulan Road, Longgang District, Shenzhen, 518112 China; 4 School of Life Sciences, Henan University, Kaifeng, 475004 China; 5 Shenzhen Research Institute of Henan University, Shenzhen, 518000 China

**Keywords:** Computational biology and bioinformatics, Genetics, Microbiology

## Abstract

Multiplex PCR is an increasingly popular method for identifying species, investigating environmental diversity, and conducting phylogenetic analysis. The complexity and increasing availability of diverse templates necessitate a highly automated approach to design degenerate primer pairs for specific targets with multiple sequences. Existing tools for degenerate primer design suffer from poor maintenance, semi-automation, low adaptability, and low tolerance for gaps. We developed PMPrimer, a Python-based tool for automated design and evaluation of multiplex PCR primer pairs for specific targets using diverse templates. PMPrimer automatically designs optimal multiplex PCR primer pairs using a statistical-based template filter; performs multiple sequence alignment, conserved region identification, and primer design; and evaluates the primers based on template coverage, taxon specificity, and target specificity. PMPrimer identifies conserved regions using Shannon’s entropy method, tolerates gaps using a haplotype-based method, and evaluates multiplex PCR primer pairs based on template coverage and taxon specificity. We tested PMPrimer using datasets with diverse levels of conservation, sizes, and applications, including *tuf* genes of Staphylococci, *hsp65* genes of Mycobacteriaceae, and 16S ribosomal RNA genes of Archaea. PMPrimer showed outstanding performance compared with existing tools and experimental validated primers. PMPrimer is available as a Python package at https://github.com/AGIScuipeng/PMPrimer.

## Introduction

The development of advanced sequencing technologies has made it easier to obtain diverse templates for specific targets, such as nucleotide sequences in NCBI^1^, 16S ribosomal RNA gene sequences in SILVA^2^, and SARS-COV-2 genome sequences in the GISAID database^3^. Based on these data, PCR-based methods are widely used for identifying species^4^, investigating environmental diversity^5^, and conducting phylogenetic analysis^6^. It is crucial to design multiplex PCR primer pairs in an unbiased manner to target specific regions of diverse templates. The complexity and rapid growth in the availability of diverse templates have made it necessary to automate the entire in silico design workflow, including data preprocessing, data analysis, primer design, and multiplex PCR primer pair evaluation^7^.

For accurate in silico design of multiplex PCR primer pairs, it is important to filter out low-quality (too short, too long, or abnormal) and redundant templates, align diverse templates using a multiple sequence alignment tool (for example, MUSCLE5^8^), identify conserved regions based on sequence similarity (such as allele frequency or Shannon’s entropy^9^), and design primers for diverse templates (for example, Primer3 software^10^). It is also important to evaluate the primers for primer dimer formation, secondary structure (hairpin formation), melting temperature (Tm), and template coverage and to evaluate in silico multiplex PCR primer pairs or amplicons for template coverage, taxon specificity, and target specificity.

Existing tools for the design of multiplex PCR primer pairs have several shortcomings, such as poor maintenance, semi-automation, low adaptability, and low tolerance of gaps (Supplementary Table S1). DECIPHER is used to design primer pairs targeting a specific group of sequences from the whole sequence set, but its R-based package is time-consuming and its web-based tool cannot currently be accessed^11^. Many tools design primers using consensus sequences while ignoring minor alleles in consensus regions, such as PrimerDesign-M^12^, openPrimeR^13^, PhyloPrimer^14^, and rprimer^15^. PrimerDesign-M and rprimer require previous multiple sequence alignment coupled with information about the intended target regions^12,15^. The rprimer tool cannot identify conserved regions and produces many candidate multiplex PCR primer pairs, which makes it almost impossible for the user to directly obtain the optimal primer pairs^15^. PhyloPrimer is used to design primers for microbial sequences and preferentially produces non-degenerate primer pairs^14^. Furthermore, most primer design tools strip gaps for convenience, especially software based on consensus sequences. Finally, tools based on the R language, such as openPrimeR^13^ and rprimer^15^, are inefficient at processing massive amounts of data.

To overcome these shortcomings and to design multiplex PCR primer pairs automatically with improved performance, we developed PMPrimer, a Python-based tool for designing multiplex primers for specific targets using diverse templates. The only necessary input file is the target sequence file in FASTA format. The greatest strengths of PMPrimer are its abilities to identify conserved regions using Shannon’s entropy method, tolerate gaps using a haplotype-based method, and evaluate multiplex PCR primer pairs based on template coverage and taxon specificity. The target specificity of primer pairs can be assessed by using BLAST^16^.

## Methods

### PMPrimer

**Figure 1:**
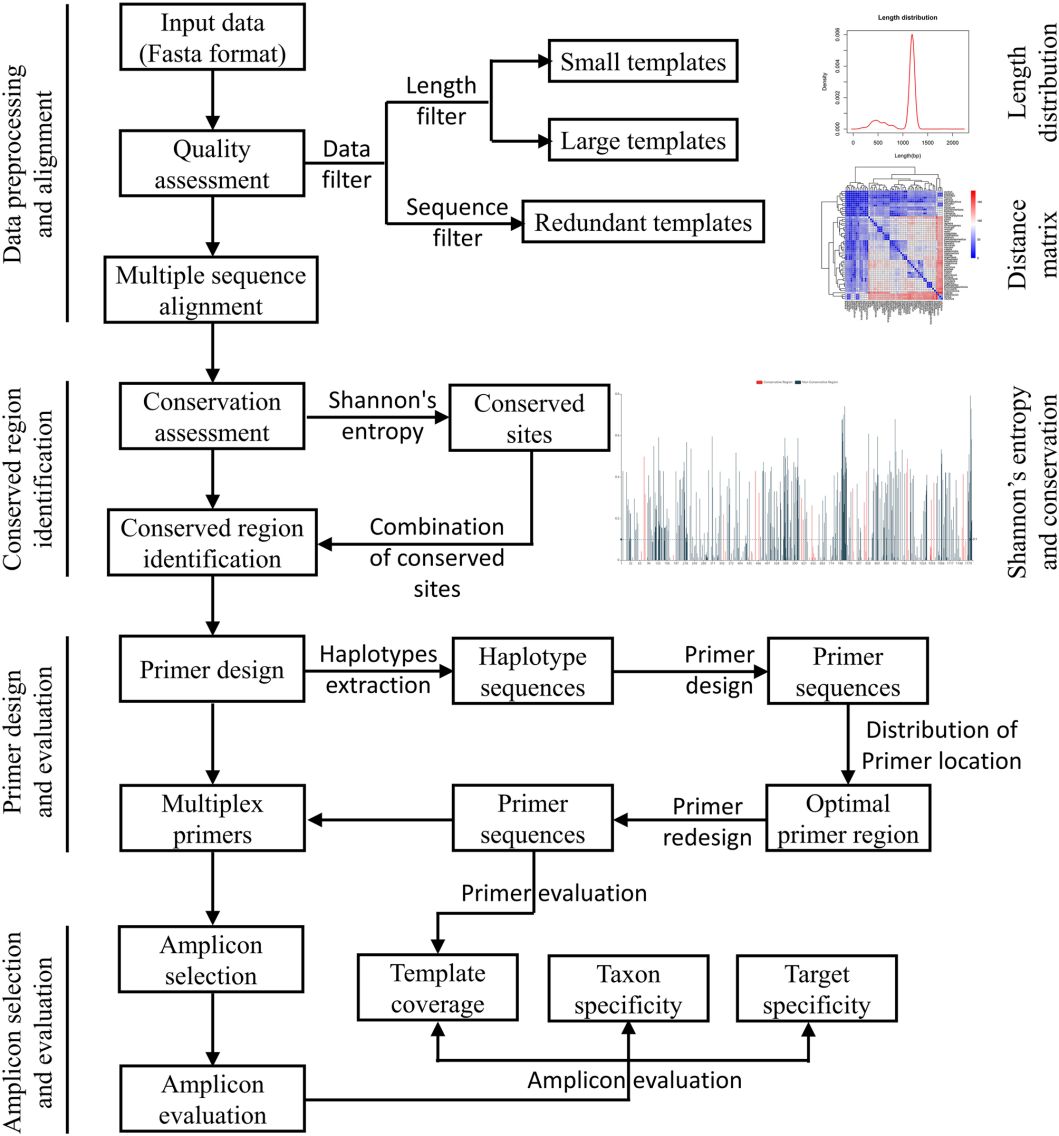
PMPrimer workflow. PMPrimer includes four main modules: data preprocessing and alignment, conserved region identification, primer design and evaluation, and amplicon selection and evaluation. The major steps are shown in the diagram.

PMPrimer is a Python-based tool for designing multiplex PCR primer pairs for specific targets using diverse templates. This tool requires MUSCLE5^8^, Primer3^10^, and BLAST^16^ and includes four major modules: data preprocessing and alignment, conserved region identification, primer design and evaluation, and amplicon selection and evaluation (Fig. 1).

#### Data preprocessing and alignment

PMPrimer starts with data quality assessment (identity duplication, record duplication, and sequence abnormality), filters out templates based on length distribution (too short or too long), and removes redundant templates with identical sequences in terminal taxa. MUSCLE5^8^ is then utilized for multiple sequence alignment. The distance matrix can be calculated based on multiple sequence alignment to estimate differences within and between taxa.

#### Conserved region identification

PMPrimer assesses the degree of complexity of nucleotide types at each position of the alignment based on Shannon’s entropy. Shannon’s entropy is calculated based on the presence of four bases (A, T, C, G) and gap symbols (–). The smaller the Shannon’s entropy value, the higher the conservation. The presence of a region with Shannon’s entropy below the desired score (default Shannon’s entropy value = 0.12) triggers the process for conserved region identification, and the length is extended as the average Shannon’s entropy drops below the desired score. To help users set a threshold Shannon’s entropy score, the minimum frequency of a major allele for a conserved position is used as the initial parameter (default major allele frequency = 0.95), and Shannon’s entropy is calculated using this threshold. The adjacent conserved regions equal to or larger than the minimum length of the initial conserved region (default = 15 bp) are then combined as the proposed conserved region. The gap positions are then counted, and the effective length of the conserved region is obtained by subtracting the gap positions from the length of the conserved region. Finally, the conserved region that satisfies the minimum length (default = 15 bp) for primer design is used for downstream analysis.

#### Primer design and evaluation

Primer design comprises five steps: (1) extracting haplotype sequences in conserved regions, (2) generating optimal primers for each haplotype sequence, (3) selecting optimal primer regions and collecting all sequences in the primer regions, (4) recomputing physicochemical properties for each primer, and (5) evaluating the template coverage of multiplex primers.

#### Amplicon selection and evaluation

Using the primers in conserved regions, multiplex PCR primer pairs are selected based on amplicon length, haplotype number limit (default = 10), and maximum difference in Tm (default = 12.0 °C). The multiplex PCR primer pairs or amplicons are then evaluated for template coverage, taxon specificity, and target specificity.

### Testing datasets

To systematically evaluate PMPrimer, we selected three datasets, 16S ribosomal RNA (rRNA) genes of Archaea, *hsp65* genes of Mycobacteriaceae, and *tuf* genes of Staphylococci, for the following reasons. First, the level of conservation varies from 3.90% similarity in 16S rRNA genes at the domain level to 89.48% similarity in *hsp65* genes at the family level to 91.73% similarity in *tuf* genes at the genus level. Second, the number of sequences in these datasets varies from thousands to tens of thousands (11,757 16S rRNA gene sequences, 6528 *hsp65* gene sequences, and 2547 *tuf* gene sequences). Third, these three types of genes are frequently used in the assessment of environmental diversity (16S rRNA genes), species identification, and clinical diagnosis (*hsp65* genes and *tuf* genes). The 16S rRNA gene is the most widely used region for bacterial taxonomy and identification in prokaryotes^17^. In mycobacteria, *hsp65* genes have higher discrimination ability for species identification compared to 16S rRNA genes and *rpoB* genes^18^. *tuf* genes are the most widely used genes for species identification of Staphylococci compared to *gap* genes, *hsp60* genes, and internal transcribed spacer regions based on searches in PubMed (https://pubmed.ncbi.nlm.nih.gov/) and Web of Science (https://www.webofscience.com) on September 7, 2023.

#### 16S rRNA genes of Archaea

We retrieved 13,421 16S rRNA genes in Archaea from the non-redundant reference dataset (SSURef 108 NR) in SILVA (release 108, accessed November 2022)^2^. After removing sequences with degenerate bases, we used the 11,757 remaining sequences as a testing dataset for 16S rRNA genes. Taxonomic classification at the phylum, class, and order levels was used to evaluate template coverage and taxon specificity in this dataset.

#### *hsp65* (*groEL2*) genes of Mycobacteriaceae

We retrieved 7188 RefSeq assemblies in the Mycobacteriaceae family from NCBI (accessed November 2022)^1^. Based on gene annotation, 12,705 possible *hsp65* gene sequences were extracted from these genomes. After low-quality and redundant sequences were filtered out, 1161 possible *hsp65* sequences remained. Gene clustering analysis was carried out based on sequence similarity, and 578 *hsp65* (*groEL2*) and 583 *groEL1* sequences were ultimately identified (Supplementary Fig. S1). Finally, 6528 *hsp65* genes corresponding to 578 non-redundant *hsp65* sequences in 221 species were used as a testing dataset.

#### *tuf* genes of Staphylococci

We retrieved 3624 *tuf* gene sequences in the *Staphylococcus* genus from the European Nucleotide Archive (ENA) of the European Bioinformatics Institute database (Coding release, accessed August 2022)^19^. The low-quality sequences (too short, too long, and redundant sequences) were filtered out, and multiple sequence alignment of the remaining sequences was performed using MUSCLE5^8^. The distance matrix was obtained based on the alignment, and unusual sequences were further filtered out. Finally, 2547 *tuf* genes from 54 species in the *Staphylococcus* genus were obtained for downstream analysis.

### Comparative analysis

To demonstrate the performance of PMPrimer, we compared this software tool with DECIPHER^11^, PrimerDesign-M^12^, PhyloPrimer^14^, rprimer^15^, and openPrimeR^13^ in terms of the analysis process, degree of automation, run time, and results. For the analysis process, modular design allows users to conveniently choose an appropriate module for subsequent analysis. Automation can be evaluated by comparing the entire analysis process and the usability of the results. Run time is an important indicator of software performance, especially in the context of big data. By comparing the template coverage and taxon specificity of the resulting multiplex PCR primer pairs, the efficiency and specificity of different software tools can be compared. The dataset for *tuf* genes from Staphylococci was used for software comparisons on Windows or WSL with Intel(R) Core(TM) i7-9700 CPU, 3.00 GHz, 16.0 GB RAM.

## Results

### PMPrimer software

PMPrimer is a Python-based tool for the automated design and evaluation of multiplex PCR primer pairs for specific targets using diverse templates (Fig. 1). To satisfy the need for automation, PMPrimer can directly extract taxonomic information at three levels of classification (genus, species, and subspecies) from input data in FASTA format downloaded from the NCBI or ENA databases. The length distribution of template sequences in the input data is calculated, and at least 90% of sequences with most common length are kept, whereas sequences that are too short or too long are removed. Redundant sequences are removed, but one representative redundant sequence is retained to preserve template diversity. For example, 578 sequences were chosen to represent 6528 *hsp65* genes of Mycobacteriaceae. Multiple sequence alignment is then performed, and the conservation of each position is scored based on Shannon’s entropy. Unlike analysis of consensus bases, Shannon’s entropy analysis preserves all types of bases, including gaps, and effectively represents the complexity of each position. Conserved regions are first identified based on Shannon’s entropy, and adjacent conserved regions are then combined, if possible; this process can tolerate interruptions caused by the presence of positions with high diversity. Furthermore, the effective length of the conserved region is obtained by subtracting gap length from the length of the conserved region. Conserved regions satisfying the minimum length requirement are used for primer design. To ensure diversity of conserved regions, all haplotype sequences are used for primer design, and the optimal primer region is selected for multiplex primers. Finally, primer pairs are selected and evaluated by electronic PCR amplification. To balance primer number and template coverage, primers are sorted and selected based on their corresponding templates. In addition, taxon specificity is evaluated using our method, which represents discrimination efficiency among different taxa at different levels of classification.

### Evaluation of PMPrimer using testing datasets

To test the performance of PMPrimer, three commonly used datasets were employed: 16S rRNA genes of Archaea, *hsp65* genes of Mycobacteriaceae, and *tuf* genes of Staphylococci. These datasets have diverse template numbers (11,757, 6528, and 2547 sequences, respectively), redundancy rates of templates (0.20%, 91.14%, and 87.63%, respectively), average similarity scores (3.90%, 89.48%, and 91.73%, respectively), gap rates in alignments (92.87%, 0.95%, and 0.58%, respectively), and taxon levels (domain, family, and genus, respectively) (Table 1).

**Table 1:** Characteristics of the three test datasets.

Dataset	Level of conservation	Average length (bp)	Number	Redundancy rate (%)	Average similarity (%)	Gap rate of alignment (%)	Taxon level
16S ribosomal RNA	Low	1147	11,757	0.20	3.90	92.87	Domain
*hsp65* genes of Mycobacteriaceae	Medium	1623	6528	91.14	89.48	0.95	Family
*tuf* genes of Staphylococci	High	1185	2547	87.63	91.73	0.58	Genus

#### 16S rRNA genes of Archaea

Based on 16S rRNA genes of Archaea, PMPrimer identified three conserved regions using the parameters “threshold for Shannon’s entropy = 0.26 (major allele frequency 0.85), gap threshold = 1.0, melting temperature = 45 °C, count of haplotype = 600” and other default parameters (Supplementary Table S2). In a previous study^5^, the best primer pair (A519F (S-D-Arch-0519-a-S-15) and 802R (S-D-Bact-0785-b-A-18)) was identified for Archaea. Here we identified one optimal degenerate primer in each of three conserved regions (Table 2). Primer 519–533 (7818–8175) is identical to previously described primer A519F, with 94.3% coverage, while primer 784–805 (10,365–11,009), with 90.4% coverage, is four bases longer than previously described primer 802R, with 90.7% coverage in our dataset. The difference between primers 784–805 and 802R is primarily due to the use of different templates (Archaea vs. Archaea and Bacteria). The 0.3% difference in coverage is due to the presence of degenerate bases in Archaea and Bacteria (in 802R, N at 789 stands for A, C, G, and T, and V at 788 stands for A, C, and G; in 784–805, H at 789 stands for A, C, and T, and S at 788 stands for C and G). Furthermore, a new primer 871–890 (12,677–13,180) was identified with 84.8% coverage in our dataset.

**Table 2:** Degenerate primers for 16S rRNA genes of Archaea.

Conserved region	Primer region	Direction	Degenerate primer	Degenerate primer count	Haplotype primer count	Coverage (%)
519-533^a^ (7818–8175)^b1^	519–533 (7818–8175)	Forward	CAGCMGCCGCGGTAA	2	2	94.3
		Reverse	TTACCGCGGCKGCTG			94.3
784–805 (10,171–11,015)^1^	784–805 (10,365–11,009)	Forward	SGGATTAGATACCCSDGTAGTC	12	10	90.4
		Reverse	GACTACHSGGGTATCTAATCCS			90.4
871–890 (11,582–13,404)^2^	871–890 (12,677–13,180)	Forward	TAARGGAATTGGCGGGRGRG	8	5	84.8
		Reverse	CYCYCCCGCCAATTCCYTTA			84.8

^a^The numbering is based on *Escherichia coli* (NC_000913.3) nomenclature.

^b^The numbering is based on the alignment.

^1^Region identified by PMPrimer and in a previous study.

^2^Region only identified by PMPrimer.

#### *hsp65 *(*groEL2*) genes of Mycobacteriaceae

*Hsp65* is frequently used for species identification in Mycobacteriaceae using primer pair Tb11 and Tb12^4,18^, which was designed 30 years ago^20^. To evaluate this primer pair and to design optimal primer pairs for mycobacteria, we built a dataset using *hsp65* gene sequences from 6 genera, 221 species, and 234 subspecies. Based on in silico evaluation, Tb11 had only 83.0% (480/578) coverage and Tb12 had only 20.4% (118/578) coverage in our dataset. PMPrimer identified nine conserved regions with the parameters “count of haplotype = 70” and other default parameters and designed one optimal degenerate primer in each of these nine conserved regions (Supplementary Table S3). For convenience, we also provide numbering based on the *hsp65* gene of *Mycobacterium tuberculosis* (NC_000962.3). In the alignment, Tb11 and Tb12 are located at positions 148–168 and 569–589, respectively. We identified two degenerate primers adjacent to Tb11 and Tb12 with higher coverage (163–181 (166–184) with 99.3% coverage and 514–531 (517–534) with 97.9% coverage). Using these degenerate primers, we identified primer pairs suitable for 300-bp fragments (short reads from Illumina and Ion Torrent sequencing), 600-bp fragments (long reads from Illumina and Ion Torrent sequencing), and > 600-bp fragments (from PacBio and NanoPore sequencing) (Table 3) for species identification and comparative analysis.

**Table 3:** Amplicons suitable for different fragment lengths in *hsp65* (*groEL2*) genes of Mycobacteriaceae.

Amplicon	Forward primer	Reverse primer	Length	Template coverage (%)	Taxon specificity		
					Genus (%)	Species (%)	Subspecies (%)
300-bp fragment length							
514–531 (517–534) to 727–755 (730–758)	GTCATCACSGTCGARGAG	CRTCYTCRGCGATGATCAG	242	94.81	100.0	82.81	78.63
600-bp fragment length							
193–209 (196–212) to 514–531 (517–534)	ACGARAAGATYGGYGC	CTCYTCGACSGTGATGAC	339	97.92	100.0	87.78	82.91
727–755 (730–758) to 1081–1097 (1084–1100)	CTGATCATCGCYGARGAYG	GYTCCTGCARYTTCTC	371	95.33	100.0	88.69	85.04
193–209 (196–212) to 727–755 (730–758)	ACGARAAGATYGGYGC	CRTCYTCRGCGATGATCAG	563	95.50	100.0	89.59	86.32
514–531 (517–534) to 1081–1097 (1084–1100)	GTCATCACSGTCGARGAG	GYTCCTGCARYTTCTC	584	97.40	100.0	91.40	88.46
> 600-bp fragment length							
193–209 (196–212) to 1081–1097 (1084–1100)	ACGARAAGATYGGYGC	GYTCCTGCARYTTCTC	905	99.13	100.0	93.21	90.60

#### *tuf* genes of Staphylococci

In an earlier study, the *tuf* gene was used for species identification of coagulase-negative Staphylococci using four primer pairs^21^. In the current study, we retrieved *tuf* gene sequences of Staphylococci from the ENA and ultimately obtained 2547 *tuf* gene sequences in this dataset, which included 54 species. Based on in silico evaluation, there was only 0.03% coverage in the original files for primer Tuf32. Therefore, we removed the amplicon produced by the Tuf32/900 primer pair from subsequent analysis. The coverage of the three other primer pairs/six primers was 100% (positions 57–77, 165–184, 339–360, 453–474, 698–717, and 835–852 in the alignment). PMPrimer identified 18 conserved regions with the parameters “threshold for Shannon’s entropy = 0.02 (major allele frequency 0.995), minimum length of initial conserved region = 5, merge = TRUE” and other default parameters (Supplementary Table S4). The six primers designed in the previous study were also identified by PMPrimer, with one base shift, one/two base differences, or divided into two primers due to highly variable positions. For convenience, we also provide numbering based on the *tuf* gene of *Staphylococcus aureus* (NC_007795.1). Based on the above degenerate primers, we also identified optimal primer pairs suitable for fragments of different lengths (Supplementary Table S5).

### Software comparison

To date, several primer design tools based on multiple sequence alignment have been developed, but each of these tools was optimized for a specific application, making it difficult to compare them. Nevertheless, we selected DECIPHER^11^, PrimerDesign-M^12^, openPrimeR^13^, PhyloPrimer^14^, and rprimer^15^ for comparisons with PMPrimer as a whole or in part to demonstrate the strengths and weaknesses of PMPrimer. PhyloPrimer could not be run due to the lack of a user manual, so we did not include it in downstream analysis. Because all these selected tools cannot generate results from datasets for 16S rRNA genes of Archaea or *hsp65* (*groEL2)* genes of Mycobacteriaceae within 12 h or exceeding the limit of the input file size, we only used the *tuf* dataset for comparison after removing redundant sequences.

PMPrimer can run with unaligned input sequences directly, rerun from any step based on modular design, and produce filtered results with evaluations for optimized primers or primer pairs. However, PrimerDesign-M and rprimer only support alignment sequences, PrimerDesign-M and openPrimeR require amplicon regions to be provided, and rprimer generates thousands of degenerate primers without selecting optimal primer pairs. Moreover, DECIPHER supports unaligned sequences and runs automatically and in a modular fashion, while all the other previously designed software tools do not. When we used the six primers with 100% coverage designed in a previous study as the true primer set^21^, PMPrimer identified all of these primers and additional novel primers, while rprimer and DECIPHER only identified 5 and 4 of these primers, respectively. To compare run times, we use multiple sequence alignment for all tools. rprimer ran the fastest, followed by PMPrimer and DECIPHER (Table 4).

**Table 4:** Comparison of different types of software.

Software	Reference regions	Newly identified regions	Run time (s)	Input original file	Modularity
PMPrimer	6/6	12	98	Yes	Yes
DECIPHER	4/6	NA^2^	1944	Yes	Yes
PrimerDesign-M	NA^1^	NA^1^	NA^1^	No	No
rprimer	5/6	NA^2^	60	No	No
openPrimeR	NA^1^	NA^1^	NA^1^	Yes	No

^1^Amplicon regions must be provided. ^2^Value is not provided.

## Discussion

Designing primer pairs for specific targets using diverse templates is essential for various applications; although several tools have been developed for this purpose, none meets all the requirements of these applications. Here, we developed PMPrimer for designing primers aimed at specific targets using diverse templates in both an automatic and modular manner to fill the gap between requirements and existing tools.

Most current tools (except for PhyloPrimer^14^) require users to prepare templates without abnormal sequences manually, a complicated and time-consuming process. PMPrimer can process multiple sequences downloaded directly from public databases such as NCBI^1^ and ENA^19^ with preprocessing analysis models. Furthermore, some tools, such as PrimerDesign-M^12^, openPrimeR^13^, and rprimer^15^, cannot identify conserved regions automatically. PMPrimer can identify conserved regions based on Shannon’s entropy^9^ and combine adjacent conserved regions to form larger conserved regions. Most existing tools design primers using consensus sequences, which ignores low-frequency alleles and gaps. By contrast, PMPrimer designs primers using haplotype sequences, which can tolerate minor alleles and gaps simultaneously. Although existing tools can theoretically be used for any target sequences, none can support big data. We examined the power of PMPrimer to design primers for diverse targets using three datasets with different template numbers, rates of sequence redundancy and similarity, gap rates of alignments, and taxon levels. Degenerate primers have been used as multiplex PCR primer pairs. However, this approach increases the redundancy of primers by using all possible combinations of degenerate bases^22^. Therefore, PMPrimer also provides haplotype primer pairs in target sequences, providing the minimal set of primer pairs for target regions. For examples, in the *tuf* dataset, the forward degenerate primer 58–78 (58–78) (5'-CACGTTGACCAYGGTAAAACD-3') represents six primers, but two of these primers do not exist; the reverse degenerate primer 334–353 (340–359) (5′-TCACGMGTTTGWGGCATTGG-3′) represents four primers, but one of these primers does not exist. Consequently, the amplicon produced by the above degenerate primer pair is theoretically produced using 24 primer pair combinations, but 12 of these do not exist. In addition, PMPrimer provides several indicators to evaluate multiplex PCR primer pairs, such as template coverage, taxon specificity, and target specificity.

To handle the diverse characteristics of target sequences, PMPrimer has multiple built-in parameters to make the appropriate adjustments for data processing. For instance, different Shannon’s entropy scores are used for target sequences with different conservation levels, such as “threshold for Shannon’s entropy = 0.26 (major allele frequency 0.85)” for 16S rRNA genes of Archaea, “threshold for Shannon’s entropy = 0.12 (major allele frequency 0.95) (default)” for *hsp65* genes of Mycobacteriaceae, and “threshold for Shannon’s entropy = 0.02 (major allele frequency 0.995)” for *tuf* genes of Staphylococci. When the target sequences are highly diverse, we recommend using “gaps parameter” to tolerate gaps and “merge parameter” to combine adjacent conserved regions.

PMPrimer has several limitations. First, it can only extract taxonomic information at the genus, species, and subspecies levels from input data automatically. We will add an analysis model to obtain taxonomic information for any continuous three level classification based on sequence description and taxonomy databases in the future. Second, some parameters must be set for specific target sequences manually. For convenience, we will automatically detect some important parameters by evaluating target sequences, such as Shannon’s entropy score and minimum allele frequency. Third, conserved region identification is the key to primer design. PMPrimer can currently identify conserved regions using specific parameters. However, we expect PMPrimer to be able to identify all possible conserved regions by iteration. Fourth, PMPrimer only provides in silico validation for newly designed primers or primer pairs. We plan to develop a tool to deal with amplicon sequencing data using information obtained during the primer design stage, such as haplotype primer pairs, distance matrix at different taxon levels, and specific positions with high variability. We will then evaluate newly designed primer pairs systematically using amplicon sequencing data.

## Conclusions

PMPrimer can be used to design and evaluate multiplex PCR primers in an automatic, modular, efficient manner using diverse templates with different characteristics. In silico evaluation using three datasets demonstrated that PMPrimer can be used for various applications.

### Supplementary Information

Supplementary Information.

## Data Availability

Datasets analyzed in this study are available at https://github.com/AGISCuipeng/PMPrimer_datasets.

## Code availability

PMPrimer software is freely available at https://github.com/AGIScuipeng/PMPrimer.
