# Supplementary material for: A tool to automatically design multiplex PCR primer pairs for specific targets using diverse templates
Source: Sci Rep. 2023 Sep 30;13:16451. doi: 10.1038/s41598-023-43825-0 (PMC10542359; doi:10.1038/s41598-023-43825-0)
Supplement: Supplementary file 1 — Supplementary Information. [file 41598_2023_43825_MOESM1_ESM.pdf]

A tool to automatically design multiplex PCR primer pairs for specific targets using diverse templates

Lin Yang[1,2+], Feng Ding[3+], Qiang Lin[1+], Junhua Xie[1,4,5], Wei Fan[1], Fangyin Dai[2*], Peng Cui[1*], Wanfei Liu[1*]

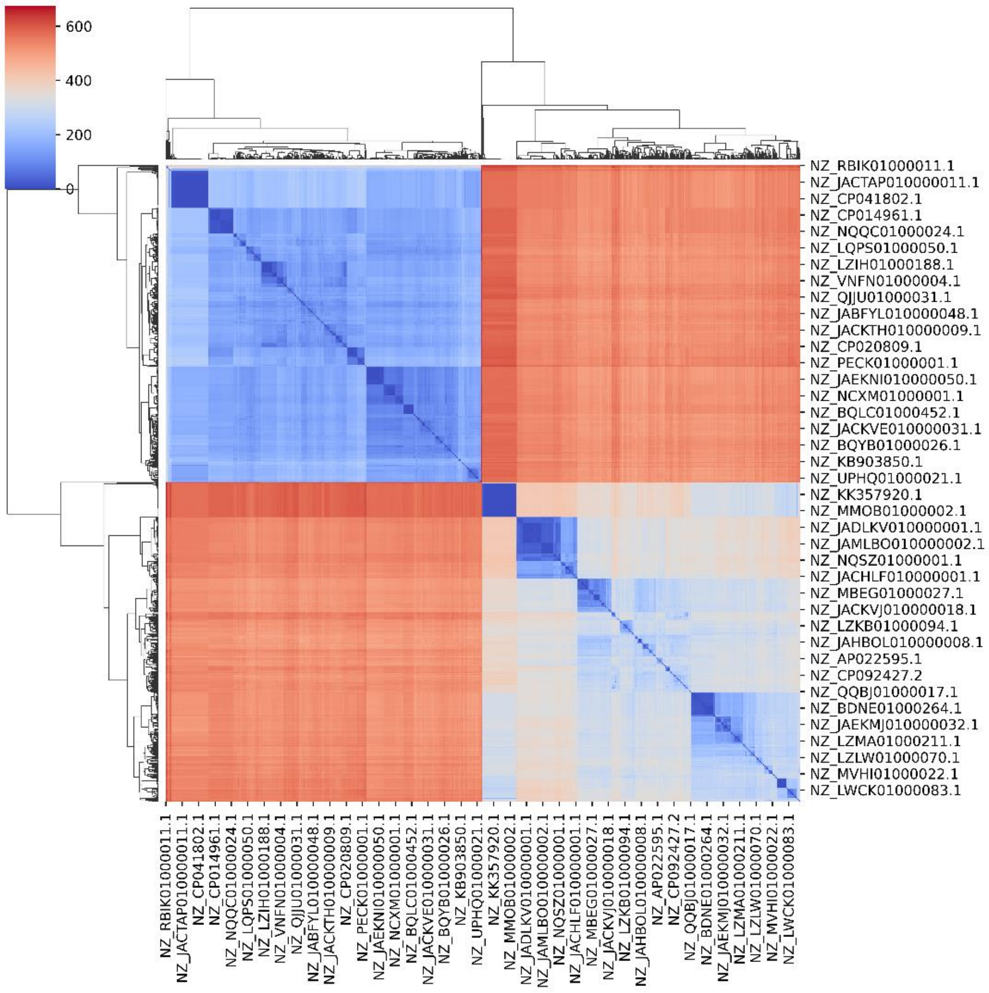

Supplementary Figure S1. The heatmap of distance matrix among suspected *hsp65* sequences in Mycobacteriaceae.

Method	Template	Specific Application	Support unaligned sequence	Target region	Primer design	Primer	Primer selection	Primer evaluation	Reference
DECIPHER	Subgroup	All	Yes	Auto selection	Consensus sequence	Degenerate primer	Yes	Template coverage	DesignSignatures: a tool for designing primers that yields amplicons with distinct signatures
PrimerDesign-M	All	All	No	Assignment	Consensus sequence	Degenerate primer	Yes	No	PrimerDesign-M: a multiple-alignment based multiple-primer design tool for walking across variable genomes
openPrimeR	All	Immunoglobulin genes	Yes	Assignment	Consensus sequence	Primer pair	Yes	Template coverage	openPrimeR for multiplex amplification of highly diverse templates
PhyloPrimer	Subgroup /All	Prokaryote	Yes	Auto selection	Consensus sequence	Normal primer	Yes	Taxon specificity & Target specificity	PhyloPrimer: a taxon-specific oligonucleotide design platform
rprimer	All	Virus	No	Auto selection	Consensus sequence	Degenerate primer	No	Template coverage	rprimer: an R/bioconductor package for design of degenerate oligos for sequence variable viruses
PMPrimer	Subgroup /All	All	Yes	Auto selection	Haplotype sequence	Degenerate primer & Primer pair	Yes	Template coverage, Taxon specificity & Target specificity	This study

Supplementary Table S1. Methods for design of multiplex PCR primers for diverse templates.

Conserved region	Primer region	Direction	Degenerate primer	Haplotype primer: count	Coverage
519-533[a](7818-8175)[b1]	519-533(7818-8175)	forward	CAGCMGCCGCGGTAA	CAGCAGCCGCGGTAA : 225	94.3%
		reverse	TTACCGCGGCKGCTG	CAGCCGCCGCGGTAA : 10861	94.3%
781-806(10171-11015)[1]	784-805(10365-11009)	forward	SGGATTAGATACCCSDGTAGTC	GGGATTAGATACCCGGGTAGTC : 23	90.4%
				GGGATTAGATACCCGAGTAGTC : 1	
				GGGATTAGATACCCGTGTAGTC : 0	
				GGGATTAGATACCCCGGTAGTC : 973	
				GGGATTAGATACCCCAGTAGTC : 47	
				GGGATTAGATACCCCTGTAGTC : 172	
		reverse	GACTACHSGGGTATCTAATCCS	CGGATTAGATACCCGGGTAGTC : 9000	90.4%
				CGGATTAGATACCCGAGTAGTC : 353	
				CGGATTAGATACCCGTGTAGTC : 51	
				CGGATTAGATACCCCGGTAGTC : 4	
				CGGATTAGATACCCCAGTAGTC : 0	
				CGGATTAGATACCCCTGTAGTC : 1	
864-893(11582-13404)[2]	871-890(12677-13180)	forward	TAARGGAATTGGCGGGRGRG	TAAGGGAATTGGCGGGGGGG : 0	84.8%
				TAAGGGAATTGGCGGGGGAG : 115	
				TAAGGGAATTGGCGGGAGGG : 0	
				TAAGGGAATTGGCGGGAGAG : 20	
		reverse	CYCYCCCGCCAATTCCYTTA	TAAAGGAATTGGCGGGGGGG : 165	84.8%
				TAAAGGAATTGGCGGGGGAG : 9530	
				TAAAGGAATTGGCGGGAGGG : 0	
				TAAAGGAATTGGCGGGAGAG : 137	

Supplementary Table S2. Degenerate and haplotype primers for 16S rRNA genes of Archaea. Notes: [a]The numbering is based on the *Escherichia coli* (NC_000913.3) system of nomenclature. [b]The numbering is based on the alignment. [1]Region identified by PMPrimer and in a previous study. [2]Region only identified by PMPrimer.

Conserved region	Primer region	Direction	Degenerate primer	Haplotype primer:count	Coverage
160-182[a](163-185)[b]	163-181(166-184)	forward	ATCGCYAAGGARATCGAVC	ATCGCTAAGGAGATCGAGC : 1	99.3%
				ATCGCTAAGGAGATCGACC : 0	
				ATCGCTAAGGAGATCGAAC : 0	
				ATCGCTAAGGAAATCGAGC : 2	
				ATCGCTAAGGAAATCGACC : 0	
				ATCGCTAAGGAAATCGAAC : 0	
		reverse	GBTCGATYTCCTTRGCGAT	ATCGCCAAGGAGATCGAGC : 550	99.3%
				ATCGCCAAGGAGATCGACC : 3	
				ATCGCCAAGGAGATCGAAC : 17	
				ATCGCCAAGGAAATCGAGC : 1	
				ATCGCCAAGGAAATCGACC : 0	
				ATCGCCAAGGAAATCGAAC : 0	
193-209(196-212)	194-209(197-212)	forward	ACGARAAGATYGGYGC	ACGAGAAGATTGGTGC : 0	100.0%
				ACGAGAAGATTGGCGC : 4	
				ACGAGAAGATCGGTGC : 16	
				ACGAGAAGATCGGCGC : 554	
		reverse	GCRCCRATCTTYTCGT	ACGAAAAGATTGGTGC : 0	100.0%
				ACGAAAAGATTGGCGC : 0	
				ACGAAAAGATCGGTGC : 0	
				ACGAAAAGATCGGCGC : 4	
514-531(517-534)	514-531(517-534)	forward	GTCATCACBGTYGARGAG	GTCATCACGGTTGAGGAG : 0	97.9%
				GTCATCACGGTTGAAGAG : 0	
				GTCATCACGGTCGAGGAG : 7	
				GTCATCACGGTCGAAGAG : 0	
		reverse	CTCYTCRACVGTGATGAC	GTCATCACTGTTGAGGAG : 0	97.9%
				GTCATCACTGTTGAAGAG : 0	
				GTCATCACTGTCGAGGAG : 4	
				GTCATCACTGTCGAAGAG : 2	

				GTCATCACCGTTGAGGAG : 3	
				GTCATCACCGTTGAAGAG : 0	
				GTCATCACCGTCGAGGAG : 542	
				GTCATCACCGTCGAAGAG : 8	
		forward	CTGATCATCGCYGARGAYG	CTGATCATCGCTGAGGATG : 0	95.5%
				CTGATCATCGCTGAGGACG : 3	
				CTGATCATCGCTGAAGATG : 0	
				CTGATCATCGCTGAAGACG : 14	
727-755(730-758)	736-754(739-757)	reverse	CRTCYTCRGCGATGATCAG	CTGATCATCGCCGAGGATG : 10	95.5%
				CTGATCATCGCCGAGGACG : 520	
				CTGATCATCGCCGAAGATG : 0	
				CTGATCATCGCCGAAGACG : 5	
		forward	GAACAGCGAYWSCGACTACG	GAACAGCGATAGCGACTACG : 0	92.4%
				GAACAGCGATACCGACTACG : 0	
				GAACAGCGATTGCGACTACG : 0	
				GAACAGCGATTCCGACTACG : 9	
1048-1079(1051-1082)	1056-1075(1059-1078)	reverse	CGTAGTCGSWRTCGCTGTTC	GAACAGCGACAGCGACTACG : 10	92.4%
				GAACAGCGACACCGACTACG : 0	
				GAACAGCGACTGCGACTACG : 0	
				GAACAGCGACTCCGACTACG : 515	
		forward	GAGAAGYTSCAGGARCG	GAGAAGTTGCAGGAGCG : 6	99.1%
				GAGAAGTTGCAGGAACG : 0	
				GAGAAGTTCCAGGAGCG : 0	
				GAGAAGTTCCAGGAACG : 0	
1081-1097(1084-1100)	1081-1097(1084-1100)	reverse	CGYTCCTGSARCTTCTC	GAGAAGCTGCAGGAGCG : 560	99.1%
				GAGAAGCTGCAGGAACG : 4	
				GAGAAGCTCCAGGAGCG : 3	
				GAGAAGCTCCAGGAACG : 0	
1099-1115(1102-1118)	1099-1113(1102-1116)	forward	YTGGCCAARCTSGCC	TTGGCCAAGCTGGCC : 5	96.4%

				TTGGCCAAGCTCGCC : 0	
				TTGGCCAAACTGGCC : 0	
				TTGGCCAAACTCGCC : 0	
		reverse	GGCSAGYTTGGCCAR	CTGGCCAAGCTGGCC : 543	96.4%
				CTGGCCAAGCTCGCC : 4	
				CTGGCCAAACTGGCC : 5	
				CTGGCCAAACTCGCC : 0	
		forward	CAAGCAYCGYATCGARGA	CAAGCATCGTATCGAGGA : 0	98.1%
				CAAGCATCGTATCGAAGA : 0	
				CAAGCATCGCATCGAGGA : 8	
1159-1187(1162-1190)	1170-1187(1173-1190)			CAAGCATCGCATCGAAGA : 0	
		reverse	TCYTCGATRCGRTGCTTG	CAAGCACCGTATCGAGGA : 17	98.1%
				CAAGCACCGTATCGAAGA : 0	
				CAAGCACCGCATCGAGGA : 519	
				CAAGCACCGCATCGAAGA : 23	
		forward	GARGGCATYGTYGCC	GAGGGCATTGTTGCC : 0	98.3%
				GAGGGCATTGTCGCC : 3	
				GAGGGCATCGTTGCC : 4	
1219-1235(1222-1238)	1219-1233(1222-1236)			GAGGGCATCGTCGCC : 549	
		reverse	GGCRACRATGCCYTC	GAAGGCATTGTTGCC : 0	98.3%
				GAAGGCATTGTCGCC : 0	
				GAAGGCATCGTTGCC : 0	
				GAAGGCATCGTCGCC : 12	

Supplementary Table S3. Degenerate and haplotype primers for *hsp65* (*groEL2*) genes of Mycobacteriaceae. Notes: [a]The numbering is based on the *hsp65* gene of *Mycobacterium Tuberculosis* (NC_000962.3). [b]The numbering is based on the alignment.

Conserved region	Primer region	Direction	Degenerate primer	Haplotype primer:count	coverage
58-89[a](58-89)[b1]	58-78(58-78)	forward	CACGTTGACCAYGGTAAAACD	CACGTTGACCATGGTAAAACG : 12	100.0%
				CACGTTGACCATGGTAAAACA : 116	
				CACGTTGACCATGGTAAAACT : 183	
		reverse	HGTTTTACCRTGGTCAACGTG	CACGTTGACCACGGTAAAACG : 0	100.0%
				CACGTTGACCACGGTAAAACA : 0	
				CACGTTGACCACGGTAAAACT : 4	
160-182(163-185)[1]	160-180(163-183)	forward	CCWGAAGAAAAAGARCGTGGT	CCAGAAGAAAAAGAGCGTGGT : 2	100.0%
				CCAGAAGAAAAAGAACGTGGT : 312	
		reverse	ACCACGYTCTTTTTCTTCWGG	CCTGAAGAAAAAGAGCGTGGT : 0	100.0%
				CCTGAAGAAAAAGAACGTGGT : 1	
223-245(226-248)[2]	223-243(226-246)	forward	CGTCACTAYGCDCACGTWGAC	CGTCACTATGCGCACGTAGAC : 0	98.4%
				CGTCACTATGCGCACGTTGAC : 48	
				CGTCACTATGCACACGTAGAC : 0	
				CGTCACTATGCACACGTTGAC : 51	
				CGTCACTATGCTCACGTAGAC : 14	
				CGTCACTATGCTCACGTTGAC : 150	
		reverse	GTCWACGTGHGCRTAGTGACG	CGTCACTACGCGCACGTAGAC : 0	98.4%
				CGTCACTACGCGCACGTTGAC : 0	
				CGTCACTACGCACACGTAGAC : 0	
				CGTCACTACGCACACGTTGAC : 0	
				CGTCACTACGCTCACGTAGAC : 0	
				CGTCACTACGCTCACGTTGAC : 47	
256-278(259-281)[2]	256-277(259-280)	forward	GCTGACTAYGTKAAAAAYATGA	GCTGACTATGTGAAAAATATGA : 0	99.4%
				GCTGACTATGTGAAAAACATGA : 12	
				GCTGACTATGTTAAAAATATGA : 8	
		reverse	TCATRTTTTTMACRTAGTCAGC	GCTGACTATGTTAAAAACATGA : 235	99.4%
				GCTGACTACGTGAAAAATATGA : 0	
				GCTGACTACGTGAAAAACATGA : 0	

				GCTGACTACGTTAAAAATATGA : 0	
				GCTGACTACGTTAAAAACATGA : 58	
292-314(298-320) [2]	296-314(302-320)	forward	TGGAYGGHGSTATCTTAGT	TGGATGGAGGTATCTTAGT : 0	98.7%
				TGGATGGAGCTATCTTAGT : 2	
				TGGATGGCGGTATCTTAGT : 5	
				TGGATGGCGCTATCTTAGT : 0	
				TGGATGGTGGTATCTTAGT : 0	
				TGGATGGTGCTATCTTAGT : 0	
		reverse	ACTAAGATASCDCCRTCCA	TGGACGGAGGTATCTTAGT : 0	98.7%
				TGGACGGAGCTATCTTAGT : 67	
				TGGACGGCGGTATCTTAGT : 172	
				TGGACGGCGCTATCTTAGT : 8	
				TGGACGGTGGTATCTTAGT : 57	
				TGGACGGTGCTATCTTAGT : 0	
334-353(340-359)[1]	334-353(340-359)	forward	CCAATGCCWCAAACKCGTGA	CCAATGCCACAAACGCGTGA : 4	100.0%
				CCAATGCCACAAACTCGTGA : 275	
		reverse	TCACGMGTTTGWGGCATTGG	CCAATGCCTCAAACGCGTGA : 0	100.0%
				CCAATGCCTCAAACTCGTGA : 36	
364-383(370-389) [2]	367-383(373-389)	forward	TCWCGTAAYGTWGGTGT	TCACGTAATGTAGGTGT : 0	99.7%
				TCACGTAATGTTGGTGT : 5	
				TCACGTAACGTAGGTGT : 22	
				TCACGTAACGTTGGTGT : 284	
		reverse	ACACCWACRTTACGWGA	TCTCGTAATGTAGGTGT : 0	99.7%
				TCTCGTAATGTTGGTGT : 0	
				TCTCGTAACGTAGGTGT : 0	
				TCTCGTAACGTTGGTGT : 3	
424-461(430-467)[1]	439-461(445-467)	forward	YTAGAATTAGTWGAAATGGAAGT	TTAGAATTAGTAGAAATGGAAGT : 166	100.0%
				TTAGAATTAGTTGAAATGGAAGT : 146	
		reverse	ACTTCCATTTCWACTAATTCTAR	CTAGAATTAGTAGAAATGGAAGT : 0	100.0%

				CTAGAATTAGTTGAAATGGAAGT : 3	
463-482(469-488)[1]	463-481(469-487)	forward	CGTGACTTATTAWSYGAAT	CGTGACTTATTAAGTGAAT : 1	93.7%
				CGTGACTTATTAAGCGAAT : 150	
				CGTGACTTATTAACTGAAT : 3	
				CGTGACTTATTAACCGAAT : 0	
		reverse	ATTCRSWTAATAAGTCACG	CGTGACTTATTATGTGAAT : 0	93.7%
				CGTGACTTATTATGCGAAT : 0	
				CGTGACTTATTATCTGAAT : 141	
				CGTGACTTATTATCCGAAT : 0	
601-617(607-623)[2]	601-617(607-623)	forward	CCAACWCCAGAMCGTGA	CCAACACCAGAACGTGA : 58	100.0%
				CCAACACCAGACCGTGA : 1	
		reverse	TCACGKTCTGGWGTTGG	CCAACTCCAGAACGTGA : 227	100.0%
				CCAACTCCAGACCGTGA : 29	
628-662(634-668)[2]	634-653(640-659)	forward	ATGATGCCWGTWGARGACGT	ATGATGCCAGTAGAGGACGT : 7	99.4%
				ATGATGCCAGTAGAAGACGT : 0	
				ATGATGCCAGTTGAGGACGT : 259	
				ATGATGCCAGTTGAAGACGT : 3	
		reverse	ACGTCYTCWACWGGCATCAT	ATGATGCCTGTAGAGGACGT : 0	99.4%
				ATGATGCCTGTAGAAGACGT : 0	
				ATGATGCCTGTTGAGGACGT : 44	
				ATGATGCCTGTTGAAGACGT : 0	
691-710(697-716) [1]	692-709(698-715)	forward	GTGTTGAACGTGGDCAAR	GTGTTGAACGTGGGCAAG : 0	100.0%
				GTGTTGAACGTGGGCAAA : 1	
				GTGTTGAACGTGGACAAG : 1	
		reverse	YTTGHCCACGTTCAACAC	GTGTTGAACGTGGACAAA : 5	100.0%
				GTGTTGAACGTGGTCAAG : 0	
				GTGTTGAACGTGGTCAAA : 308	
772-791(781-800)[2]	772-789(781-798)	forward	GGTGTWGARATGTTCCGT	GGTGTAGAGATGTTCCGT : 0	100.0%
		reverse	ACGGAACATYTCWACACC	GGTGTAGAAATGTTCCGT : 238	100.0%

				GGTGTTGAGATGTTCCGT : 4	
				GGTGTTGAAATGTTCCGT : 73	
		forward	GGTGCDYTATTACGWGG	GGTGCGTTATTACGAGG : 0	98.7%
				GGTGCGTTATTACGTGG : 40	
				GGTGCGCTATTACGAGG : 0	
				GGTGCGCTATTACGTGG : 0	
				GGTGCATTATTACGAGG : 1	
826-842(835-851)[1]	826-842(835-851)			GGTGCATTATTACGTGG : 151	
		reverse	CCWCGTAATARHGCACC	GGTGCACTATTACGAGG : 0	98.7%
				GGTGCACTATTACGTGG : 0	
				GGTGCTTTATTACGAGG : 0	
				GGTGCTTTATTACGTGG : 122	
				GGTGCTCTATTACGAGG : 0	
				GGTGCTCTATTACGTGG : 1	
		forward	CGTCAYACDCCWTTCTTC	CGTCATACGCCATTCTTC : 19	99.4%
				CGTCATACGCCTTTCTTC : 0	
				CGTCATACACCATTCTTC : 63	
				CGTCATACACCTTTCTTC : 0	
				CGTCATACTCCATTCTTC : 100	
955-972(964-981)[2]	955-972(964-981)			CGTCATACTCCTTTCTTC : 0	
		reverse	GAAGAAWGGHGTRTGACG	CGTCACACGCCATTCTTC : 7	99.4%
				CGTCACACGCCTTTCTTC : 0	
				CGTCACACACCATTCTTC : 2	
				CGTCACACACCTTTCTTC : 0	
				CGTCACACTCCATTCTTC : 120	
				CGTCACACTCCTTTCTTC : 2	
		forward	TTYCGTACTACWGAYGTAAC	TTTCGTACTACAGATGTAAC : 0	99.7%
991-1016(1000-1025)[2]	997-1016(1006-1025)			TTTCGTACTACAGACGTAAC : 0	
		reverse	GTTACRTCWGTAGTACGRAA	TTTCGTACTACTGATGTAAC : 0	99.7%
				TTTCGTACTACTGACGTAAC : 1	

				TTCCGTACTACAGATGTAAC : 0	
				TTCCGTACTACAGACGTAAC : 12	
				TTCCGTACTACTGATGTAAC : 16	
				TTCCGTACTACTGACGTAAC : 285	
		forward	CCAGAAGGHAYWGAAATGGT	CCAGAAGGCACTGAAATGGT : 2	99.0%
				CCAGAAGGTACTGAAATGGT : 252	
				CCAGAAGGAACTGAAATGGT : 2	
				CCAGAAGGCATTGAAATGGT : 0	
				CCAGAAGGTATTGAAATGGT : 4	
1030-1052(1039-1061)[2]	1033-1052(1042-1061)			CCAGAAGGAATTGAAATGGT : 0	
		reverse	ACCATTTCWRTDCCTTCTGG	CCAGAAGGCACAGAAATGGT : 0	99.0%
				CCAGAAGGTACAGAAATGGT : 52	
				CCAGAAGGAACAGAAATGGT : 0	
				CCAGAAGGCATAGAAATGGT : 0	
				CCAGAAGGTATAGAAATGGT : 0	
				CCAGAAGGAATAGAAATGGT : 0	
		forward	GACGTACWGTWGGWTCAGGCG	GACGTACTGTTGGTTCAGGCG : 0	96.2%
				GACGTACAGTTGGTTCAGGCG : 0	
				GACGTACTGTAGGTTCAGGCG : 7	
1138-1166(1147-1175)[2]	1142-1162(1151-1171)			GACGTACAGTAGGTTCAGGCG : 0	
		reverse	CGCCTGAWCCWACWGTACGTC	GACGTACTGTTGGATCAGGCG : 96	96.2%
				GACGTACAGTTGGATCAGGCG : 15	
				GACGTACTGTAGGATCAGGCG : 185	
				GACGTACAGTAGGATCAGGCG : 0	

Supplementary Table S4. Degenerate and haplotype primers for *tuf* genes of Staphylococci. Notes: [a]The numbering is based on the the *tuf* gene of *Staphylococcus aureus* (NC_007795.1). [b]The numbering is based on the alignment. [1]Region identified by PMPrimer and in a previous study. [2]Region only identified by PMPrimer.

Amplicon	Forward primer	Reverse primer	Length	Coverage	Taxon-specificity		
					Genus	Species	Subspecies
300-bp fragment length							
334-353(340-359) to 601-617(607-623)	CCAATGCCWCAAACKCGTGA	TCACGKTCTGGWGTTGG	284	100%	100%	98.11%	98.11%
58-89(58-89) to 334-353(340-359)	CACGTTGACCAYGGTAAAACD	TCACGMGTTTGWGGCATTGG	296	100%	100%	90.57%	90.57%
600-bp fragment length							
58-89(58-89) to 424-461(430-467)	CACGTTGACCAYGGTAAAACD	TCACGWACTTCCATTTCWACT	404	100%	100%	96.23%	96.23%
160-182(163-185) to 601-617(607-623)	GAAGAAAAAGARCGTGGT	TCACGKTCTGGWGTTGG	458	100%	100%	98.11%	98.11%
334-353(340-359) to 772-791(781-800)	CCAATGCCWCAAACKCGTGA	ACGGAACATYTCWACACC	458	100%	100%	98.11%	98.11%
58-89(58-89) to 601-617(607-623)	CACGTTGACCAYGGTAAAACD	TCACGKTCTGGWGTTGG	560	100%	100%	98.11%	98.11%
>600-bp fragment length							
160-182(163-185) to 772-791(781-800)	GAAGAAAAAGARCGTGGT	ACGGAACATYTCWACACC	632	100%	100%	98.11%	98.11%
58-89(58-89) to 772-791(781-800)	CACGTTGACCAYGGTAAAACD	ACGGAACATYTCWACACC	734	100%	100%	98.11%	98.11%

Supplementary Table S5. Amplicons suitable for different fragment lengths in *tuf* genes of Staphylococci.
